# Evaluation of various levels of sweet almond meal as a source of protein on the production variables and immune response of broiler chickens

**DOI:** 10.1002/vms3.386

**Published:** 2020-11-07

**Authors:** Zeinab Moradi Yeganeh, Somayyeh Salari, Khalil Mirzadeh, Mohsen Sari, Mohammadreza Ghorbani

**Affiliations:** ^1^ Department of Animal Science, Animal Science and Food Technology Faculty Agricultural Sciences and Natural Resources University of Khuzestan Ahvaz Iran

**Keywords:** carcass characteristic, immunity, lipid variables, performance

## Abstract

This study was conducted to evaluate effects of a sweet almond meal (SAM) as a source of protein on performance and immune responses of broiler chickens. Treatments consisted of different levels of SAM (7%, 14%, 21% and 28% of diet) and control diet that was performed in a completely randomized design in 42 days. Body weight gain (BWG) and feed intake (FI) were recorded weekly. Carcass characteristics and blood variables were measured on the 42 day of age. Immune response to sheep red blood cell (SRBC) was assessed at d 35 and d 42 (7 days after each injection). White blood cell count was done at d 28. The results showed that chickens fed 28% of SAM had lowest FI during the experiment. During grower period and also whole period of experiment, BWG was highest in chickens that consumed 7% SAM. Feed conversion ratio (FCR) significantly increased in chickens fed 28% SAM compared to the other treatments during grower period and also whole period of the experiment. The relative weight of thigh and breast significantly decreased and the relative weight of GIT increased by increasing levels of SAM in the diet. The concentration of cholesterol and LDL significantly decreased in chickens that consumed SAM in the diet. However, the highest glucose and HDL concentration were observed in chickens fed 28% SAM in the diet. Treatments did not have a significant effect on the immune response of broilers (primary and secondary SRBC antibody titres, relative weights of spleen and bursa of fabricius and white blood cell count) (*p* > 0.05). The results of this study show that a diet supplemented with SAM at the level of 14%, due to the improved FCR and decreased blood cholesterol and LDL concentration, can be a good replacement for soybean meal in broiler chicken's nutrition.

## Graphical abstract

This experiment was conducted to evaluate effects of various levels of sweet almond meal (SAM) as source of protein in broiler nutrition with different levels of SAM (0, 7%, 14%, 21% and 28% of diet). The results of this study show that a diet supplemented with SAM at the level of 14%, due to the improved FCR and decreased blood cholesterol and LDL concentration, can be a good replacement for soybean meal in broiler chicken's nutrition.

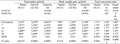

## 1 INTRODUCTION

Due to COVID‐19 crisis and drastic changes in the feed chain and feed supply, the search for alternative feed supplies becomes urgently apparent and needs further research (Hafez & Attia, 2020). Also, soybean meal is the main protein source in poultry diets. The international prices of this feedstuff have increased the costs of poultry production, and therefore, reduced marketing margins. Additionally, due to the high amount of wastes from the agricultural sector and food industry, proper management of these resources and identifying the nutritional value, makes it possible to produce cheap and suitable ingredients for poultry which are not competitive with human food (Al‐Harthi et al., 2019, 2020). Agroindustry wastes from tomato, olive, date and sunflower processing have valuable nutritional characteristics, and could be included in poultry rations to facilitate reducing feeding costs (Seidavi et al., 2018). By‐products of tomato and olive processing can be supplemented in the diet of broilers up to 10% without detrimental effects on production, while improving the biochemical blood profile of chickens. It was shown olive cake is a valuable ingredient for inclusion in broilers' pelleted diet up to 15% during 7–28 days of age with phytase supplementation (Al‐Harthi et al., 2020). Also Al‐Harthi et al. (2019) showed that Prosopis pods meal could be used as an alternative feedstuff for poultry production, specially, in tropical and sub‐tropical regions where feed resources are generally limited. Al‐Sagan et al. (2020) reported dietary addition of 30% blue lupine with the addition of probiotics could provide a cheap source of protein without negative effects on the growth performance, carcass characteristics, immune organs and gut morphology of broilers. It has been shown that feeding sunflower and date wastes at high inclusion levels (up to 30% and 40%, respectively) resulting in heavier broiler carcasses (Seidavi et al., 2018). Also, it has been reported that citrus and grape wastes can be successfully included in broilers diets up to 3%, which increases carcass and meat yield and reduces abdominal fat (by 10% on average). Pomegranate can be included up to 2% supplementation and is associated with improved feed efficiency (by 12% in broilers) (Azizi et al., 2018). Moreover, the use of these agricultural by‐products, such as almond fruit meal, in poultry feeding can help to reduce waste and environmental pollution concerns. Almond tree, botanically known as *Terminalia catappa* tree, originated from tropical Asia, India, the Malay Peninsula and Taiwan. Almond nuts (*Prunus amygdalus*) can be grouped into: (1) sweet almonds (*Prunus amygdalus dulcis*) and (2) bitter almonds (*Prunus amygdalus amara*) (Monaghan, 2008). According to FAO (2012), the top four almond producers in the world were the United States, followed by Spain, Australia and Iran. The almond production in the world has a growing rate of 5% annually. Almond with 87,708 hectares of cultivated area is one of the most important horticulture products in Iran (FAO, 2012). According to USDA (2013), almond nut contains dry matter, ether extract, crude protein, ash and crude fibre at a concentration 953, 494.2, 212.2, 29.9 and 122 g/kg, respectively. Oil extraction of sweet almond is done by cold pressing methods, so sweet almond meal (SAM) contains considerable amounts of oil. Although the role of dietary inclusion of cashew nut by‐products in broiler chickens have been investigated in the past (Freitas et al., 2006), there is a lack of concrete knowledge about the SAM in poultry nutrition. Freitas et al. (2006) reported that the cashew bran consists of fragments of almonds (i.e. cashew seeds) which contain high energy value due to the presence of high fat and high quantities of protein (22%–38%), and can replace partially with corn and soybean meal in the broiler feed. However, it also contains anti‐nutritional factors, most specifically tannic acid, which can be harmful to the performance of broiler chickens (Ramos et al., 2006). It was demonstrated that the use of almond and its by‐products can decrease blood LDL concentration and increase HDL concentration (Jahanban‐Esfahlan et al., 2010) in humans. Also, whole almond seed, brown skin, shell and hull possess potent free radical scavenging capacities (Amarowicz et al., 2005; Jahanban‐Esfahlan et al., 2010; Moure et al., 2007). It has been reported that due to the presence of fibre in almond skins, almond meal can alter the composition of gut bacteria and can act as potential prebiotics in human diets (Mandalari et al., 2008). However, the literature lacks a thorough study on the effect of almond by‐products in the broiler chick nutrition. Therefore, we evaluate the effects of SAM as a novel feedstuff on the performance and blood variables of broiler chickens.

## 2 MATERIALS AND METHODS

All the procedures undertaken in our study were approved by the Animal Ethics Committee at Agricultural Sciences and Natural Resources University of Khuzestan, Ahvaz, Iran.

### 2.1 Preparation and analysis of sweet almond meal

Sweet almond meal was prepared from the Barij Essence Pharmaceutical Company, Kashan, Iran. Four samples of SAM were prepared and analysed for dry matter (DM), crude protein (CP), crude fibre (CF), ether extract (EE) and ash according to AOAC (2002). The results are shown in Table 1.

**TABLE 1:** Chemical composition of sweet almond meal (%)

Dry matter	Crude protein	Ether extract	Crude fibre	Ash	Phosphorous	NFE^a^	ME^b^
96	48	11	8.70	4.51	0.822	23.8	2,930

^a^ Nitrogen free extract = 100 − (Moisture + CP + EE + CF + Ash).

^b^ Metabolizable energy value was calculated using an equation from NRC (soybean meal, expeller) (Janssen, 1989) (kcal/kg).

### 2.2 Birds, diets and general procedures

One‐day‐old male chicks (Ross 308) were obtained from a local hatchery, housed in floor pens, and fed according to a standard broiler diet for week 1. Feed and water were provided ad libitum for their consumption. On day 8, totally 220 chickens were weighed individually and distributed randomly into five treatment groups with four replicates (11 chicks per replicate) for each treatment group in a completely randomized design. Treatments consisted of different levels of SAM (7%, 14%, 21% and 28% of diet) and control diet (without SAM). The feeding regimen consisted of a starter (for day 8–21), and grower (for day 22–42) diet that was formulated to meet the nutrient requirements of broilers according to NRC (1994). All diets were prepared freshly every week and diets were in mash form. Feed and water were provided ad libitum throughout the experiment. The ingredients and chemical composition of the diets are shown in Tables 2 and 3. All the birds were vaccinated with Newcastle disease virus (NDV) vaccine on days 3 and 8 of age and also vaccinated against Gamboro at day 14 of age. Light intensity was kept constant continuously for the first 3 days of post‐hatching, after which a 23L:1D lighting schedule was maintained during the experiment. At day 1 of age, the temperature was set at 33°C and subsequently was reduced by 2°C/week.

**TABLE 2:** Composition and nutrient contents of the basal diet and diets with increasing levels of sweet almond meal (SAM) in the starter phase (d 8 to 21 posthatch)

Ingredients	Level of SAM (%)				
	Control	7	14	21	28
Corn	54.9	55.6	56	58.7	60.8
Soybean meal (44% crude protein)	36.8	30.3	23.5	14.7	6
SAM (48% crude protein)	0	7	14	21	28
Fish meal (60% crude protein)	2	1	0	0	0
Vegetable oil	2.5	2	2	0.7	0
Limestone	1.27	1.3	1.34	1.34	1.24
Dicalcium phosphate	1.4	1.61	1.8	2	2.05
Salt	0.45	0.47	0.47	0.47	0.47
Vitamin premix^a^	0.25	0.25	0.25	0.25	0.25
Mineral premix^b^	0.25	0.25	0.25	0.25	0.25
DL‐Methionine	0.14	0.17	0.21	0.23	0.26
L‐Lysine	0	0	0.1	0.35	0.63
Chemical composition (%)					
AME (kcal/kg)	3,045	3,045	3,045	3,045	3,045
Crude protein	21.9	22	22	22	22.1
Ether extract	4.58	5.22	5.86	5.38	5.48
Crud fibre	2.67	2.97	3.27	3.54	3.87
Calcium	1	1	1	1	1
Available phosphorous	0.45	0.45	0.45	0.45	0.45
Sodium	0.2	0.2	0.2	0.2	0.2
Lysine	1.41	1.23	1.1	1.1	1.1
Methionine + Cystine	0.9	0.9	0.9	0.9	0.9

^a^ Supplied per kilogram diet: vitamin A (retinyl acetate), 9,000 IU; vitamin D3, 3,000 IU; vitamin E (DL‐α‐tocopheryl acetate), 48 mg; vitamin K, 3 mg; thiamin, 1.8 mg; riboflavin, 6 mg; pyridoxine, 3 mg; vitamin B12, 0.012 mg; niacin, 42 mg; folic acid, 1.2 mg; biotin, 0.24 mg; pantothenic acid, 12 mg.

^b^ Supplied per kilogram of diet: manganese, 120 mg; zinc 100 mg; iron, 80 mg; copper, 20 mg; iodine, 2 mg; selenium, 0.3 mg; cobalt, 0.5 mg.

**TABLE 3:** Composition and nutrient contents of the basal diet and diets with increasing levels of sweet almond meal (SAM) in the grower phase (d 22 to 42 posthatch)

Ingredients	Level of SAM (%)				
	Control	7	14	21	28
Corn	61.5	60.6	61.1	63.8	66.8
Soybean meal (44% crude protein)	29.6	25	17.8	8.83	0
SAM (48% crude protein)	0	7	14	21	28
Fish meal (60% crude protein)	3	1	0	0	0
Bicarbonate sodium	0.07	0.11	0.15	0.15	0.15
Vegetable oil	3	3	3	2	0.5
Limestone	1.3	1.33	1.33	1.29	1.27
Dicalcium phosphate	0.75	1.16	1.4	1.5	1.6
Common salt	0.25	0.25	0.25	0.25	0.25
Vitamin premix^a^	0.25	0.25	0.25	0.25	0.25
Mineral premix^b^	0.25	0.25	0.25	0.25	0.25
DL‐ Methionine	0.03	0.1	0.16	0.21	0.27
L‐Lysine	0	0	0.17	0.42	0.67
Chemical composition (%)					
AME (kcal/kg)	3,173	3,173	3,173	3,173	3,173
Crude protein	20	20.1	20	20	20.1
Ether extract	6.79	7.32	7.01	6.81	7.56
Crud fibre	2.52	2.86	3.17	3.43	3.68
Calcium	0.9	0.9	0.9	0.9	0.9
Available phosphorous	0.35	0.35	0.35	0.35	0.35
Sodium	0.15	0.15	0.15	0.15	0.15
Lysine	1.26	1.08	1	1	1
Methionine + Cystine	0.73	0.73	0.73	0.73	0.73

^a^ Supplied per kilogram diet: vitamin A (retinyl acetate), 9,000 IU; vitamin D3, 3,000 IU; vitamin E (DL‐α‐tocopheryl acetate), 48 mg; vitamin K, 3 mg; thiamin, 1.8 mg; riboflavin, 6 mg; pyridoxine, 3 mg; vitamin B12, 0.012 mg; niacin, 42 mg; folic acid, 1.2 mg; biotin, 0.24 mg; pantothenic acid, 12 mg.

^b^ Supplied per kilogram of diet: manganese, 120 mg; zinc 100 mg; iron, 80 mg; copper, 20 mg; iodine, 2 mg; selenium, 0.3 mg; cobalt, 0.5 mg.

### 2.3 Production variables

Weekly body weight gain (BWG) and feed intake (FI) of each pen were recorded. Feed conversion ratio (FCR) was calculated by dividing FI by BWG.

### 2.4 Size of different organs

At the end of the experiment, two chickens from each replicate (which were close to the mean of BW of the replicate) were selected and slaughtered to evaluate the relative weights (based on BW) of the breast, thigh, gastrointestinal tract, liver, gizzard and abdominal fat. Also lymphoid organs (spleen and bursa of fabricius) were weighed and calculated as a percentage of live BW.

### 2.5 Antibody titre and white blood cell count

At 28 and 35 days of age, two chickens from each replicate were given a single injection of 1 ml of a 10% suspension of sheep red blood cell (SRBC) via the brachial vein. Seven days after each injection, a sample of approximately 0.5 ml of blood was obtained from the brachial vein of each individual and transferred into a tube containing two drops of ethylenediaminetetra‐acetic acid (EDTA). After refrigeration (needed for the red blood cells to settle), plasma antibodies were measured by the microtitre haemagglutination method of Wegmann and Smithies (1966). Titres were expressed as log2 of the reciprocal of the highest dilution in which there was haemagglutination.

At 28 days of age, blood samples from wing veins were taken from two chicks of each replicate. The smears were stained using May Grunwald‐Giemsa stain. The heterophils and lymphocytes were counted to a total of 60 cells (Gross & Siegel, 1983) and were examined by light microscopy. The results are presented as the percentage of each cell occurring in each film. The ratio of heterophil/lymphocyte (H/L) was examined by dividing the number of heterophils by the number of lymphocytes.

### 2.6 Blood lipid metabolites

At the end of the study (42 days), blood samples were collected via the brachial vein of two chickens per replicate. The serum content was separated after centrifugation at 4,500 *g* and 4°C for 10 min, and kept at −20°C for further analysis. Serum samples were analysed for concentrations of low‐density lipoprotein (LDL), high‐density lipoprotein (HDL), cholesterol and triglycerides (TG) using standard kits (Zist Shimi, Tehran, Iran) and with an autoanalyser (Autolab PM 4000; Medical System, Rome, Italy).

### 2.7 Statistical analysis

Data were analysed in a completely randomized design using the General Linear Model procedures of SAS Institute (2001). The following model was assumed in the analysis of all studied traits.

$$Y_{\text{ij}}=\mu +T_{i}+\varepsilon_{\text{ij}}$$

where, Y_ij_ is the observed value for a particular character, µ is the overall mean, T_i_ is the effect of the i^th^ treatment and ε_ij_ is the random error associated with ij^th^ recording. Data were analysed considering the pen of chickens as the experiment unit for the performance variables. Individual chickens were measured as experimental units for the rest of the variables. Significant differences among the treatments were determined using Tukey's test at *p* < 0.05.

## 3 RESULTS

### 3.1 Production variables

The data representing the effect of dietary inclusion of SAM on the performance of broilers are shown in Table 4. The mortality was lower than 2% with no differences observed between the groups. From day 8 to day 21, dietary inclusion of 28% of SAM into the diet caused a decrease (*p* < 0.05) in FI and BWG of chickens compared to other treatments. From day 22 to day 42 and also from day 8 to day 42, FI and BWG were decreased (*p* < 0.05) by the inclusion of 28% SAM into the diet compared to other treatments. The best FCR was observed in the chickens that consumed 7% and 14% of SAM which had a significant difference compared to the other treatments at the starter period. However, the FCR improved significantly (*p* < 0.05) in chickens fed 7% and 14% of SAM diet than those fed 21% and 28% of SAM (*p* < 0.05) during the whole period of the experiment.

**TABLE 4:** Effect of various levels of sweet almond meal (SAM) on performance of broiler chickens

Level of SAM (%)	Feed intake (g/bird)			Body weight gain (g/bird)			Feed conversion ratio		
	Starter (8–21)	Grower (22–42)	Experiment overall (8–42)	Starter (8–21)	Grower (22–42)	Experiment overall (8–42)	Starter (8–21)	Grower (22–42)	Experiment overall (8–42)
0 (Control)	1,015^a^	3,078^b^	4,093^ab^	593^b^	1,745^b^	2,198^b^	1.71^a^	1.82^b^	1.86^cb^
7	1,071^a^	3,282^a^	4,353^a^	696^a^	2,058^a^	2,614^a^	1.54^b^	1.64^b^	1.66^d^
14	1,023^a^	3,232^ab^	4,255^a^	640^a^	1,886^b^	2,387^a^	1.60^b^	1.77^b^	1.78^cd^
21	1,000^ab^	3,093^b^	3,994^b^	564^b^	1,657^b^	2,081^b^	1.77^a^	1.86^b^	1.92^b^
28	884^b^	2,698^c^	3,582^c^	485^c^	1,337^c^	1,683^c^	1.82^a^	2.09^a^	2.12^a^
*SEM*	16.3	29.5	34.6	15.1	23	20.2	0.034	0.048	0.02
*p*‐value	0.0133	0.0001	0.0001	0.319	0.0001	0.0001	0.044	0.0002	0.0001

^a–d^Means within column with different superscripts differ significantly (*p* < 0.05).

### 3.2 Size of different organs

**FIGURE 1:**
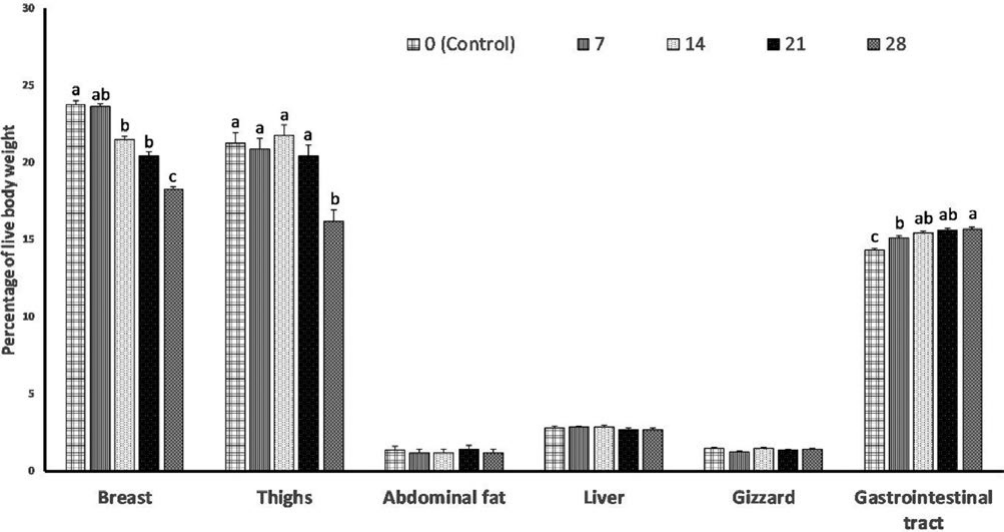
Effects of various levels of sweet almond meal (SAM) on carcass characteristics of broiler chickens at 42 days of age. Different letters (a‐c) indicate a significant difference between treatment groups (*p* < 0.05). Data are shown as mean ± *SEM*

There were no effects (*p* > 0.05) of different levels of SAM on the relative weights (% of BW) of the gizzard, liver and abdominal fat (Figure 1). Relative weights of the thigh and breast were decreased (*p* < 0.05) as SAM levels increased in the diets. However, the relative weight of the gastrointestinal tract was increased (*p* < 0.05) as dietary levels of SAM increased from 7% to 28%.

### 3.3 Sheep red blood cell antibody responses

Treatments did not have a significant effect on primary and secondary SRBC antibody titres of broilers (*p* > 0.05). The relative weights of spleen and bursa of fabricius were not influenced by the treatments (*p* > 0.05; Table 5).

**TABLE 5:** Effect of various levels of sweet almond meal (SAM) on anti‐SRBC^1^ antibody levels, relative weight of immune organs (% of live body weight) and percentage of white blood cells of broiler chickens in 42 days of age

Level of SAM (%)	Anti‐SRBC antibody levels, log_2_						
	Primary	Secondary	Bursa of fabricius	Spleen	Lymphocyte	Heterophile	H/L
0 (Control)	6	9.5	0.04	0.06	59.75	38.5	0.652
7	5.75	10.25	0.05	0.05	58	39.5	0.686
14	6.25	9.75	0.03	0.05	62	37.75	0.612
21	6	9.25	0.03	0.08	57.5	41.5	0.731
28	5.5	10	0.02	0.06	56.5	41	0.728
*SEM*	0.42	0.19	0.004	0.005	0.951	0.828	0.024
*p*‐value	0.761	0.524	0.316	0.663	0.416	0.606	0.526

Abbreviation: SRBC, Sheep red blood cell.

### 3.4 White blood cell

There were no effects (*p* > 0.05) of different levels of SAM on the percentage of white blood cells and H/L ratio (Table 5).

### 3.5 Blood lipid metabolites

**FIGURE 2:**
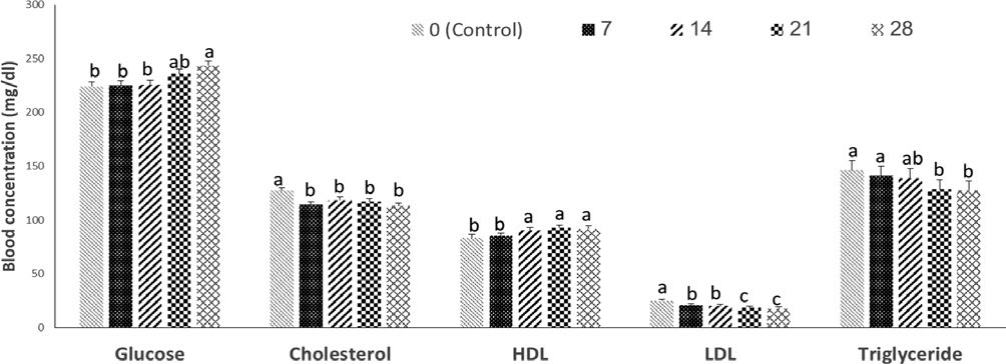
Effects of various levels of sweet almond meal (SAM) on blood variables of broiler chickens at 42 days of age. Different letters (a‐c) indicate a significant difference between treatment groups (*p* < 0.05). Data are shown as mean ± *SEM*

As shown in Figure 2, dietary inclusion of SAM, caused a significant decrease in the plasma cholesterol levels (*p* < 0.05). Also, the plasma HDL level of chickens fed with SAM (14%–28%) diets was significantly higher than that of those fed 7% SAM and control diet (*p* < 0.05). However, blood triglyceride and LDL concentrations of chickens that fed 21% and 28% of SAM significantly decreased compared to those of chickens that consumed control diet (*p* < 0.05). Also, dietary inclusion of 28% of SAM, caused a significant (*p* < 0.05) increase in the plasma glucose levels compared to that in other treatment groups, except for the 21% SAM diet.

## 4 DISCUSSION

According to the results, the growth performances of the chickens fed diets with different levels of SAM up to 21% were not statistically different compared to those in the control group. However, the chickens fed with the diet containing 28% of SAM showed significantly lower FI and BWG and higher FCR compared to the control diet. There is no report in the literature regarding the effect of SAM on broiler chickens' performance. According to the research of Oliveira (2018), the use of by‐product in the diet of broilers at the final phase was not recommended because they decrease body weight gain of the chickens. It has been shown that the presence of lipids in the almond nut meal can affect the cholecystokinin release, increase pancreatic secretion, act on the satiety centre and inhibit FI (Bertechini, 2013). On the other hand, tannins found in almond meal affect the taste buds and proteolytic enzymes, which can trigger a negative feedback on digestive physiology and the animal's satiety centre, interrupting FI to prevent further damage to the body (Kamath & Rajini, 2007). Therefore, the reduction of performance variables of the chickens at 28% of SAM, possibly due to the anti‐nutritional effect of the fibre fraction of SAM on the intestinal motility and viscosity (Arruda et al., 2012). Also, this reduction may be related to the effect of phytates on the availability of mineral elements, and the negative effect of condensed tannins which are known to affect FI of animals by inhibiting the activities of digestive enzymes and reducing the absorptive capacity of feed (Glahn et al., 2002). Oliveira (2018) reported dietary inclusion of some ingredients like sorghum, because of tannin content, maybe increased the feed conversion ratio of broiler chicks. Consistent with our results, Viveros et al. (2011) observed that dietary inclusion of grape extract (rich in polyphenols like tannin) at a high level (7.2 g/kg) reduced the weight gain by 12%. Chamorro et al. (2019) showed that the dietary incorporation of grape extract up to 2.5 g/kg in chicken diets did not affect the growth performance, whereas a dose of 5 g/kg impaired the growth rate (by 5%) and digestibility of some essential and non‐essential amino acids, with a particularly marked reduction for proline. Although, Freitas et al. (2011) reported that up to 24% of the almond bran of cashew nut can be added in diets of broilers (1–21 days). Also, in contrast to our findings, Arjomandi et al. (2015) reported no difference in performance variables of Japanese quails fed with sweet almond supplements at the levels of 0, 10, 20 and 30%. Annongu et al. (2005) showed that treated fermented almond fruit waste with enzyme improved FI, BWG and FCR of cockerels whereas untreated almond fruit waste elicited poor performance and high mortality of the chickens. Freitas et al. (2006) reported cashew nut bran at the levels of 0, 5, 10, 15, 20 and 25% of diet, did not influence the feed consumption of broiler chickens. However, the increase in cashew nut bran in the feed promoted a linear increase in weight gain, at all stages, and a linear improvement in feed conversion ratio, both in the initial phase and in the total period of experiment. Also, Ramos et al. (2006) reported that dietary inclusion of cashew pulp at the levels of 0, 5, 10 and 15%, did not influence FI and BWG of broiler chickens. Apata and Atteh (2016) reported that broilers fed the fermented almond fruit meal supplemented with *Aspergillus niger*, had higher (*p* < 0.05) average daily weight gains, average FI at the starter, grower and whole period of the experiment compared to those on unfermented and control diets. However, the FCR was improved in chickens that consumed fermented almond fruit meal compared with the unfermented only at the starter period. Sateri et al. (2017) examined the effects of different levels of olive meal (0, 2, 4, 6 and 8%) and enzymatic supplements (mixture of β‐glucanase, phytase and hemicellulase) in the diet of broiler chickens. Their results showed that for a 42‐day feeding period, there were no differences between the body weight, feed intake and feed conversion ratio among treatments.

Dietary inclusion of SAM decreased the relative weights of the thigh and breast and increased relative weight of the gastrointestinal tract. The increased weight of the gastrointestinal tract may be partially attributed to the amount of the fibre in the diet that produces physical distension of the walls of the intestinal tract. This is consistent with the reports of Apata and Atteh (2016), who found broilers fed the unfermented almond fruit meal in the diet, had a small intestinal weight higher than those fed fermented and control diets. Hetland and Svihus (2001) found that high fibre diets of oat hulls generated intestinal distension, increased gastrointestinal capacity and gut fill in broiler chickens. The weight, volume and capacity of rat gastrointestinal tract were shown to increase with increasing dietary fibre (Hansen et al., 1992). Apart from this, an increase in the weight of gastrointestinal tract observed in our experiment could also reflect the impact of tannic acid in the SAM which corroborates the report of Mansoori et al. (2007), who found that dietary tannic acid increased the relative weight of the intestine of broiler cockerels. In contrast to our findings, Arjomandi et al. (2015) reported no differences in the relative weight of different internal organs and carcass traits at 42 days of age of Japanese quails by supplementing the diet with SAM. Sayehban et al. (2015) showed that adding 10% of olive meal to the diet of broiler chickens increased the relative weight and length of the jejunum. In this study, the relative weight of breast and thighs was decreased in chickens fed 28% of SAM which is in accordance with the performance results. There was a decline in the BWG of broiler chickens fed 28% SAM. Although, the relative weight of thighs increased in groups fed up to 21% of SAM. Similarly, Starčević et al. (2015) demonstrated that the supplementation of tannic acid to broiler diets markedly increased the fat percentage in breast and thigh muscles.

As shown in Table 5, primary and secondary SRBC antibody titres were not affected by the treatments. Also, there was no effect of different SAM levels on the relative weight of spleen, bursa of fabricius percentage, white blood cells count and H/L ratio. Al‐Khalifa et al. (2012) reported that the thymus, spleen and bursa of fabricius are the main immune organs in poultry. During an immune response, mature lymphocytes and other immune cells interact with antigens in these tissues. Consequently, in some cases immune tissue development can indicate immune system response. Cazaban and Gardin (2012) showed that in young chickens the bursa of fabricius plays an important role in boosting immunity. Also, it was demonstrated polyphenols can improve immune response in domestic animals, which seems to be through three mechanisms. First, these compounds can enhance the proliferation of beneficial bacteria, while reducing the abundance of pathogenic ones through their bactericidal and bacteriostatic activities, thus indirectly improving host immunity and health (Gessner et al., 2017). Second, these molecules possess antioxidant capacity and can counteract the free radical generated during the immune response and heat stress, thereby improving the animal's immune system (Lipiński et al., 2016). Third, polyphenols are also able to positively regulate the production of cytokines, heat shock proteins and transcription factors (Lipiński et al., 2016). In this study, SAM did not influence the immune variables of broilers, which could be due to an insufficient amount of polyphenols in this by‐product. Sateri et al. (2017) examined the effect of different levels of olive meal (0, 2, 4, 6 and 8% of diet) in broiler chickens. Their results showed that antibody titres, after vaccination against infectious bronchitis virus and Gumboro disease, were higher in chickens fed 4% olive meal.

Dietary inclusion of SAM caused a decrease in plasma cholesterol, TG and LDL levels and an increase in plasma glucose and HDL levels of broilers. To our knowledge, there is no study that evaluated the effect of dietary inclusion of SAM on broiler blood lipid variables. It was shown that higher dietary fibre content reduced dietary fat utilization by deconjugation of bile salts (Story & Furumoto, 1990; Story & Kritchevsky, 1976), which could reduce fat absorption through the gut, therefore, the body fat (liver fat) could be utilized for the metabolic needs and increased the HDL concentration in serum. The reduced triglyceride concentration in the serum of broilers fed high levels of SAM (21% and 28% of diet) also supports this hypothesis. A similar trend was observed in the experiments of Rama Rao et al. (2004), Rama Rao et al. (2006), in which the serum concentrations of LDL and TG decreased in chickens receiving high‐fibre diets. Hosseini‐Vashan and Raei‐Moghadam (2019) reported the concentration of cholesterol and LDL decreased when the levels of pomegranate pulp gradually increased from 0% to 10% of diet. They proposed that antioxidant components in pomegranate pulp have a lowering effect on blood lipids. Our results showed a good agreement with those obtained by other studies such as Arjomandi et al. (2015), who found that quails fed diets containing 20% and 30% SAM had a blood cholesterol level significantly lower than those fed diets containing 10% SAM and control. Also, they reported that quails fed diets containing 20% and 30% SAM had a LDL level significantly lower than those fed control diet. Similarly, Fulgoni et al. (2002) showed that almond nut in the human diet can reduce blood total cholesterol and LDL while increasing blood HDL. These effects of almonds are hypothesized to be mediated by components in the oil fraction of almond nut or probably in part because of the non‐fat (protein and fibre) and monounsaturated fatty acid components of the nut (Jenkins et al., 2002). Elson (1995) found that isoprenoids can suppress cholesterol synthesis by inhibiting the production of 3‐hydroxy‐3‐methylglutaryl coenzyme A (HMG‐CoA) reductase, which is the rate‐controlling enzyme of the cholesterol synthetic pathway.

## 5 CONCLUSION

Here, we showed that supplementing broiler chickens' diets with SAM at the level of 14%, improved FCR and reduced blood cholesterol and LDL concentrations. Our findings can also be insightful for the broiler chicken's nutrition industry, as we showed that SAM supplementations can be potentially used as a replacement for the soybean meal in poultry nutrition.

## CONFLICTS OF INTEREST

All authors approve the submission of this manuscript and declare no conflict of interest. The manuscript has not been published previously, and not under consideration for publication elsewhere.

## AUTHOR CONTRIBUTION

Zeinab Moradi Yeganeh: Data curation; Investigation. Somayyeh Salari: Conceptualization; Project administration; Supervision; Writing‐review & editing. Khalil Mirzadeh: Methodology. Mohsen Sari: Formal analysis. Mohammadreza Ghorbani: Data curation.

### PEER REVIEW

The peer review history for this article is available at https://publons.com/publon/10.1002/vms3.386.
